# Clinical Characteristics and Survival of Hepatocellular Carcinoma: Insights from Single-Centre Experience in Saudi Arabia

**DOI:** 10.7759/cureus.52608

**Published:** 2024-01-20

**Authors:** Ahmed M Badheeb, Mohammed K Al Sedran, Faisal Ahmed, Ibrahim K Al Sidran, Mohammed H Al Qurayshah, Abdullah Abu Bakar, Hamoud Y Obied, Islam A Seada, Abdelaziz Aman, Mohamed Badheeb

**Affiliations:** 1 Oncology, King Khalid Hospital, Oncology Center, Najran, SAU; 2 Emergency Medicine, King Khalid University Hospital, Najran, SAU; 3 Urology, Ibb University, Ibb, YEM; 4 Surgery, Najran University, Najran, SAU; 5 College of Medicine, Najran University, Najran, SAU; 6 Ophthalmology, King Khalid Hospital, Najran, SAU; 7 Cardiac Surgery, King Khalid Hospital, Najran, SAU; 8 Cardiothoracic Surgery, King Khalid Hospital, Najran, SAU; 9 Internal Medicine, King Khalid University Hospital, Nagran, SAU; 10 Internal Medicine, Yale New Haven Health, Bridgeport Hospital, Bridgeport, USA

**Keywords:** cirrhosis, survival, alpha-fetoproteins, saudi arabia, najran, liver neoplasm, hepatitis b virus, hepatocellular carcinoma

## Abstract

Background

Hepatocellular carcinoma (HCC) represents the most common primary liver malignancy, with a high fatality rate. Relatively, Saudi Arabia has a high incidence of HCC, which is detected in later stages with a poor prognosis. This study aims to investigate the patterns, outcomes, and mortality predictors of HCC in Saudi Arabia.

Method

A retrospective study from April 2018 to June 2022 included patients with HCC who were diagnosed and managed at the Najran Oncology Center, Saudi Arabia. Through our cancer registry, the patients' clinical, laboratory, radiological, and survival profiles were extracted and analyzed to assess factors associated with mortality using a univariate analysis. The overall survival was calculated by the Kaplan-Meier method.

Results

The study involved 52 patients with an average age of 74.6 years, predominantly male (the male-to-female ratio is 2.25:1). Viral infections were the primary cause of liver disease in 40.3% (n=21) of patients. At diagnosis, the Child-Pugh class distribution included 23.1% (n=12) patients in class A, 36.5% (n=19) patients in class B, and 40.4% (n=21) patients in class C. Uninodular tumors with ≤50% liver extension were observed in 65.4% (n=34) of cases, and 30.8% (n=16) had portal vein thrombosis. Elevated alpha-fetoprotein (AFP) levels were noted in 48.1% (n=25) of patients, with 23.1% (n=12) exceeding 400 ng/mL. Curative resection was performed in 32.7% (n=17) of patients. The mean survival time was 23±11.8 months (median of 22.5 months, minimum of six, and maximum of 49 months). Relapse occurred in seven (13.5%) cases, while new metastasis occurred in 20 (38.5%) cases. During the study period, 26 (50.0%) patients died. The main cause of death was disease progression in 15 (28.8%) patients. Univariate analysis showed that AFP>400 ng/mL (OR: 4.68; 95% CI: 1.87-11.66, p=0.001), presence of relapse (OR: 0.16; 95% CI: 0.03-0.78, p=0.023), abdominal ascites (OR: 3.38; 95% CI: 1.25-9.14, p=0.016), advanced the Cancer of the Liver Italian Program (CLIP) score (OR: 0.60; 95% CI: 0.41-0.88, p=0.009) were associated with higher mortality rate and were statistically significant.

Conclusion

Most cases of HCC in our patients were attributed to viral hepatitis, with the majority having liver cirrhosis. Higher AFP (>400 ng/mL), relapse, abdominal ascites, and a higher cancer CLIP score were associated with poorer outcomes. Targeted screening and health education should be advocated; in addition, social determinants should be proactively addressed.

## Introduction

Hepatocellular carcinoma (HCC) represents a grave outcome of chronic liver disease. Notably, chronic inflammatory alterations were observed in the majority of HCC cases [1]*.* The necro-inflammatory processes related to specific viral etiologies, primarily hepatitis B and C, and their capacity to induce genomic modifications are crucial to HCC tumorigenesis and progression. Moreover, other contributing factors, including excessive alcohol consumption and non-alcoholic steatohepatitis (NASH), are intricately linked to HCC development [2]. The incidence, progression, and demographic patterns of HCC show substantial geographical variation. African regions, for instance, reported a younger age of HCC onset compared to European nations. On the other hand, while balanced gender distributions have been observed in countries like Uganda and Ecuador, a male predominance incidence was reported in Europe [3].

Despite a regional decline in HCC cases, the Kingdom of Saudi Arabia (KSA) has witnessed an increase in age-standardized incidence rate, from 4.5 to 5.2, and age-standardized mortality rate, from 4.2 to 5.1, during a two-year timeframe from 2018 to 2020 [4]. Additionally, within the KSA region, there is a significant sub-regional diversity, with the highest HCC prevalence observed in Riyadh, Najran, and Tabuk [5]. Despite widespread screening and vaccination efforts, chronic infections with hepatitis B virus (HBV) and hepatitis C virus (HCV) continue to be the primary contributors to HCC in KSA, as suggested by reports [6]. However, there is an increasing trend in other etiologies, such as non-alcoholic steatohepatitis (NASH). 

HCC maintains an unfavorable prognosis, with a five-year overall survival rate falling below 19% [7]. Various prognostic scoring systems, including the albumin-bilirubin (ALBI) grade and the Cancer of the Liver Italian Program (CLIP) score, have been introduced to predict mortality and prognosis. However, the notable heterogeneity in the factors employed by each system may constrain their predictability and accuracy [8,9]. HCC persists as a growing national health threat in KSA, underscoring the imperative for comprehensive assessment and thorough research to bridge existing knowledge gaps and integrate a multidisciplinary approach into its management [10]. Here, our objective is to explore the etiological, clinical, and laboratory features of HCC in a singular cancer center in KSA. Additionally, we aim to pinpoint potential predictors of mortality within our patient cohort.

## Materials and methods

Study design

HCC patients who were diagnosed and managed at Najran Oncology Cancer Centre over a four-year period from April 2018 to June 2022 were included in this retrospective study. Ethics Research Committees of King Khalid Hospital approved the study (number: H-11-N-081), ensuring adherence to the principles of the Declaration of Helsinki.

All adult patients (≥18 years old) diagnosed with HCC and managed at our cancer center were included. Exclusions comprised patients with unconfirmed diagnoses or those not aligned with the recommendations of the American Association for the Study of Liver Diseases (AASLD) [11]. Additionally, patients with concurrent neoplasms alongside HCC, secondary liver malignancy due to metastasis, those incapable or unwilling to provide informed consent, individuals untraceable during the follow-up period, or those treated or referred to external institutions were excluded.

Diagnosis of HCC

HCC diagnosis relied on either AASLD guidelines or histological evaluation [11]. To establish the diagnosis of HCC, one of the following criteria should be fulfilled: (1) hepatic dysmorphological nodularity with arterial-phase contrast enhancement on CT or MRI, (2) alpha-fetoprotein (AFP) levels exceeding 200 ng/mL along with an identified liver nodule, or (3) histopathological examination.

Definitions

Patients were categorized as "cirrhotic" based on histopathological specimen examination, clinical indicators (e.g., ascites), biochemical markers of hepatic dysfunction (serum bilirubin, liver enzymes, including alanine and aspartate aminotransferase, serum alkaline phosphatase, gamma-glutamyl transpeptidase, prothrombin time, and albumin levels), along with sonographic evidence of portal hypertension [12,13]. A tumor was considered surgically amenable (i.e., treatment-eligible) if it was a single lesion smaller than 5 cm without vascular invasion or up to three lesions, each less than 3 cm without associated portal vein thrombosis. Similarly, tumors larger than 5 cm were also considered eligible for surgical resection in the absence of vascular invasion. Notably, other curative treatments, including liver transplantation, percutaneous ethanol ablation, or radio-frequency ablation, were not considered for the treatment-eligible cohorts. As a baseline assessment, the CLIP score, evaluating various tumor characteristics and cirrhosis severity by assigning scores from 0 to 6, was calculated. This score is based on four variables: the Child-Pugh class, tumor morphology, portal vein thrombosis, and serum concentration of AFP. Furthermore, the CLIP score was used to categorize the tumor morphology on the basis of nodularity (uninodular or multinodular) and extension, with a tumor's extension of >50% considered as "massive" [14].

Data collection

Using patients' medical records, collected data included demographics (age, gender), clinical presentation, identified liver etiology, and associated illnesses or risk factors. Additionally, patients were stratified based on hepatic dysfunction using the Child-Pugh score. Furthermore, initial AFP levels and the presence of portal vein thrombosis were included in the initial analysis as prognostic parameters.

Main outcome

The primary outcome of the study was identifying HCC patients' overall survival, while the secondary outcome was to investigate the factors linked to mortality in HCC cases.

Statistical analysis

Mean and standard deviation were used for descriptive data representation. We integrated either a two-sample t-test or the Mann-Whitney test for comparative analysis of quantitative data, while Fischer's exact test was utilized for qualitative data. The survival was assessed via the Kaplan-Meier model, the log-rank test, and the receiver operating characteristic (ROC). The outcome was deemed statistically significant with a p-value <0.05. IBM SPSS version 18 software (IBM Corp., Armonk, US) was used for the mentioned analyses.

## Results

The study cohort had a mean age of 74.6±13.0 years, with a range of (46-108) years and a male-to-female ratio of 2.25:1 (36 males and 16 females). The etiology of liver disease was primarily viral in 21 patients (40.3%), with HBV and HCV infections present in 21.2% and 19.2% of patients, respectively. Other etiological entities included NASH (11.5%), autoimmune hepatitis (11.5%), primary biliary cirrhosis (11.5%), alcohol abuse (5.8%), and various other causes, such as hemochromatosis and tyrosinemia, collectively accounting for 19.2% of cases. The predominant comorbid conditions were hypertension (90.4%), diabetes (57.7%), ischemic heart disease (23.1%), thyroid disease (11.5%), and renal failure (7.7%). Abdominal pain (63.5%) and jaundice (59.6%) were the most prevalent symptoms, with a mean duration from symptom onset to diagnosis of more than three months in 73.1% of the cases and under three months in 26.9%. Table 1 summarizes the characteristics of the included patients.

**Table 1:** Characteristics of hepatocellular carcinoma patients HCV - hepatitis C virus; HBV - hepatitis B virus; NASH - nonalcoholic steatohepatitis

Variables	n (%)
Age (years), mean±SD	74.6±13.0
Gender	
Female	16 (30.8%)
Male	36 (69.2%)
Cause of hepatocellular cancer	
HBV	11 (21.2%)
HCV	10 (19.2%)
NASH	6 (11.5%)
Autoimmune hepatitis	6 (11.5%)
Primary biliary cirrhosis	6 (11.5%)
Alcoholic hepatitis	3 (5.8%)
Other causes	10 (19.2%)
Meantime from symptoms to diagnosis	
More than three months	38 (73.1%)
Less than three months	14 (26.9%)
Comorbidities	
Ischemic heart disease	12 (23.1%)
Diabetes	30 (57.7%)
Hypertension	47 (90.4%)
Renal failure	4 (7.7%)
Thyroid disease	6 (11.5%)
Main symptoms	
Abdominal pain	33 (63.5%)
Gastrointestinal bleeding	10 (19.2%)
Ascites	19 (36.5%)
Jaundice	31 (59.6%)
Weight loss	15 (28.8%)

HCC diagnosis

MRI was utilized to establish HCC diagnosis in all cases. Notably, a triphasic computerized tomography scan proved diagnostic in 54% of cases, while liver biopsy was necessary in 15% of the patients. Among 19 patients (36.5%), the diagnosis was established during the evaluation and follow-up of liver cirrhosis, with a mean duration of 23±11.8 months (median of 22.5 months, minimum six and maximum 49 months). Among 16 patients (30.8%), portal vein thrombosis was identified. The AFP was high at initial diagnosis in 25 (48.1%) cases and was higher than 400 ng/mL in 12 (23.1%) cases.

Liver disease severity and tumor characteristics

HCC was diagnosed in 51 (98%) of cases who had liver cirrhosis. At the time of diagnosis establishment, Child-Pugh class A was identified in 12 (23.1%) patients, B in 19 (36.5%), and C in 21 (40.4%) patients (Table 2).

**Table 2:** Liver disease severity and tumor characteristics

Variables	n (%)
Alpha-fetoprotein	
<200 ng/mL	27 (51.9%)
200-400 ng/mL	13 (25.1%)
>400 ng/mL	12 (23.1%)
Child-Pugh class at the time of diagnosis	
A	12 (23.1%)
B	19 (36.5%)
C	21 (40.4%)
Tumor morphology	
Uninodular and extension ≤50%	34 (65.4%)
Multinodular and extension ≤50%	2 (3.8%)
Massive or extension > 50%	16 (30.8%)

Eligibility for curative treatment

The number of patients with a tumor considered eligible for curative treatment according to tumor size and its multifocality was 31 (59.6%). However, only 17 (32.7%) underwent curative resection. This treatment included transarterial chemoembolization (TACE) in six (11.5%) cases, treatment with tyrosine kinase inhibitors in four (7.7%) cases, hepatic resection or radiofrequency ablation (RFA) in six (11.5%) cases, and immunotherapy with nivolumab in one (1.9%) case. Seven patients (13.5%) underwent additional treatment modalities, including hepatic resection or radiofrequency ablation.

Patient outcome

The mean survival duration was 23±11.8 months. Relapse occurred in 13.5% of cases, and new metastases developed in 38.5%. By the end of the study, 50.0% of the patients had died. Disease progression was the leading cause of death in 15 (28.8%) cases, followed by sepsis in seven (13.5%) cases, and hepatorenal syndrome in five (9.6%) cases, as indicated in Table 3.

**Table 3:** Characteristics of hepatocellular carcinoma patients' outcome

Variables	n (%)
Final status	
Death	26 (50.0%)
Alive	26 (50.0%)
Relapse	7 (13.5%)
Cause of death	
Hepatorenal syndrome	5 (9.6%)
Disease progression	15 (28.8%)
Sepsis	7 (13.5%)

Factors associated with increased mortality

Univariate analysis showed that older age, AFP more than 400 ng/ml, higher Child-Pugh class, higher CLIP score, and presence of metastasis were highly associated with nonsurvivors and were statistically significant (all p<0.05; Table 4).

**Table 4:** Compression between survival and non-survival patients ^1 ^Linear Model ANOVA; ^2 ^Pearson's Chi-squared test ^* ^Risk factors include male gender, hepatitis C virus, hepatitis B virus, and alcohol use. CLIP score - Cancer of the Liver Italian Program scoring

Variables	Total (n=52)	Live (n=26)	Death (n=26)	p-value
Age (year), mean±SD	73.4±11.1	70.0±11.4	76.8±10.0	0.0251
Gender				1.0002
Female	16 (30.8%)	8 (30.8%)	8 (30.8%)	
Male	36 (69.2%)	18 (69.2%)	18 (69.2%)	
Nationality				0.1592
Saudi	42 (80.8%)	19 (73.1%)	23 (88.5%)	
Non-Saudi	10 (19.2%)	7 (26.9%)	3 (11.5%)	
Alpha-fetoprotein				0.0482
<400 ng/ml	40 (76.9%)	23 (88.5%)	17 (65.4%)	
>400 ng/ml	12 (23.1%)	3 (11.5%)	9 (34.6%)	
Portal vein thrombosis				0.5482
No	36 (69.2%)	17 (65.4%)	19 (73.1%)	
Yes	16 (30.8%)	9 (34.6%)	7 (26.9%)	
Tumor morphology				0.1532
Uninodular and extension ≤50%	24 (46.2%)	9 (34.6%)	15 (57.7%)	
Massive or extension >50%	16 (30.8%)	11 (42.3%)	5 (19.2%)	
Multinodular and extension ≤50%	12 (23.1%)	6 (23.1%)	6 (23.1%)	
Child-Pugh class				0.0012
A	12 (23.1%)	4 (15.4%)	8 (30.8%)	
B	19 (36.5%)	5 (19.2%)	14 (53.8%)	
C	21 (40.4%)	4 (15.4%)	17 (65.4%)	
CLIP score				0.0341
Mean (SD)	1.8 (1.2)	1.5 (1.1)	2.2 (1.2)	
Range	0.0 - 5.0	0.0 - 5.0	0.0 - 4.0	
Risk factors^*^				0.7061
Mean (SD)	1.2 (0.7)	1.1 (0.5)	1.2 (0.9)	
Range	0.0 - 3.0	0.0 - 2.0	0.0 - 3.0	
Metastasis				0.0442
No	33 (63.5%)	13 (50.0%)	20 (76.9%)	
Yes	19 (36.5%)	6 (23.1%)	13 (50.0%)	
Relapse				0.2232
No	45 (86.5%)	24 (92.3%)	21 (80.8%)	
Yes	7 (13.5%)	2 (7.7%)	5 (19.2%)	
Curative treatment receives				0.7682
No	35 (67.3%)	17 (65.4%)	18 (69.2%)	
Yes	17 (32.7%)	9 (34.6%)	8 (30.8%)	

Survival analysis and associated factors

**Figure 1:**
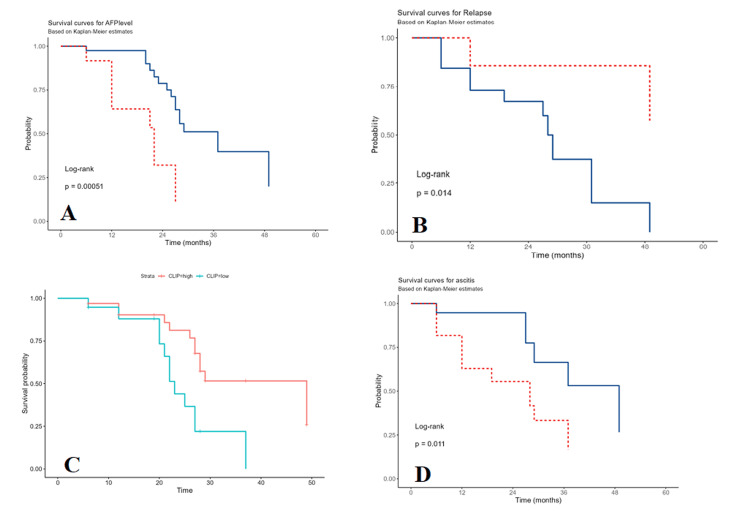
Kaplan-Meier and cumulative survival rate in patients with hepatocellular carcinoma A: by alpha-fetoprotein (AFP) levels; B: relapse; C: Cancer of the Liver Italian Program (CLIP) score; D: abdominal ascites

In the univariate analysis, AFP levels greater than 400 ng/mL (OR: 4.68, 95% CI: 1.87-11.66, p=0.001; Figure 1A), relapse (OR: 0.16, 95% CI: 0.03-0.78, p=0.023; Figure 1B), high CLIP score (OR: 0.60, 95% CI: 0.41-0.88, p=0.009; Figure 1C), and the presence of abdominal ascites (OR: 3.38, 95% CI: 1.25-9.14, p=0.016; Figure 1D) demonstrated statistically significant associations with mortality, as outlined in Table 5. Additionally, patients classified as Child-Pugh class B exhibited a 1.21 times higher risk of death compared to those in class A. Moreover, patients with Child-Pugh class C faced a 2.81 times higher risk of death than those in class A, although this disparity did not achieve statistical significance

**Table 5:** Factors associated with survival analysis CLIP score: Cancer of the Liver Italian Program scoring

Variables	Subgroups	n (%)	OR (95% CI)	p-value
Gender	Female	16 (30.8)	Reference value	0.548
	Male	36 (69.2)	0.77 (0.34-1.78)	
Nationality	Saudi	42 (80.8)	Reference value	0.446
	Non-Saudi	10 (19.2)	1.62 (0.47-5.64)	
Alpha-fetoprotein	<400 ng/mL	40 (76.9)	Reference value	0.001
	>400 ng/mL	12 (23.1)	4.68 (1.87-11.66)	
Portal vein thrombosis	Absent	36 (69.2)	Reference value	0.335
	Present	16 (30.8)	0.64 (0.26-1.59)	
Tumor morphology	Uninodular	24 (46.2)	Reference value	
	Massive (>50%)	16 (30.8)	2.36 (0.96-5.76)	0.060
	Multinodular	12 (23.1)	1.30 (0.45-3.78)	0.624
Metastasis	No	33 (63.5)	Reference value	0.124
	Yes	19 (36.5)	1.83 (0.85-3.97)	
Child-Pugh class	A	12 (23.1)	Reference value	
	B	19 (36.5)	1.21 (0.32-4.61)	0.777
	C	21 (40.4)	2.81 (0.94-8.40)	0.064
Relapse	No	45 (86.5)	Reference value	0.023
	Yes	7 (13.5)	0.16 (0.03-0.78)	
Age	Mean±SD	73.4±11.1	1.04 (1.00-1.09)	0.072
CLIP score	Mean±SD	1.8±1.2	0.60 (0.41-0.88)	0.009
Time to diagnosis	<3 months	38 (73.1)	Reference value	0.514
	≥3 months	14 (26.9)	1.37 (0.53-3.52)	
Ascites	No	19 (36.5)	Reference value	0.016
	Yes	33 (63.5)	3.38 (1.25-9.14)	

## Discussion

The national and global burden of HCC is immense, with over 900 thousand cases diagnosed worldwide in 2020 and an overall five-year survival ranging from 17% to 21% [15,16]. Within KSA, the age-standardized incidence rate (ASIR) increased from 4.5 to 5.2 between 2018 and 2020 [17]. Despite HCC ranking as the eighth most incident malignancy in KSA, it stands as the primary cause of cancer-related mortality [16]. Remarkably, Najran exhibits a notably higher incidence of 10/100,000 within KSA regions [17].

HCC disproportionately affects older males, typically between 50 and 60 years old, aligning with findings from prior studies [6,18]. In our investigation, the mean age at the time of diagnosis was 74 years, predominantly affecting male patients (69.2%) with a male-to-female ratio of 2.25:1. This pattern is consistent with earlier reports in KSA [6,19]. The underlying factors contributing to this gender disparity are not fully elucidated, though metabolic differences, including a higher incidence of diabetes, visceral adiposity, and behavioral risks such as alcohol consumption, may play a role. Furthermore, elevated rates of HBV and HCV infections among males may contribute to this gender-based variation [6]. 

In our study, viral infections constituted the etiology of HCC in 40.3% of cases, with HCV more predominant than HBV (19.2% vs. 21.2%). This observed trend deviates from prior reports, where HBV traditionally accounted for the majority of viral-related HCC [20]. Despite reports indicating an overall decline in viral hepatitis incidence, HBV continues to prevail, underscoring the significance of vaccination programs. The higher incidence of HCV observed in our study may reflect an increased propensity for HCC development among HCV patients rather than an actual rise in prevalence [21,22]. Irrespective, viral hepatitis remains a substantial health threat in KSA. Mitigating the escalating trend of viral hepatitis necessitates concerted efforts, including targeted education and awareness campaigns for the public. Counseling on disease transmission and natural history is crucial. Moreover, initiatives must address cultural acceptance, aiming to reduce stigma and discrimination against individuals with HBV or HCV. Such efforts facilitate early-stage treatment associated with a higher likelihood of recovery [10,23].

An overwhelming majority of our patients, constituting approximately 98%, developed HCC on the background of liver cirrhosis. Consistent with prior reports, where over 80% of HCC cases were associated with liver cirrhosis, our findings underscore the strong correlation between HCC and cirrhotic conditions [24]. Nonetheless, it is noteworthy that HCC occurrences devoid of cirrhosis have been documented, often identified at advanced stages with poorer outcomes attributed to the lack of regular follow-up [25]. In our cohort, HCC emerged during the follow-up of liver cirrhosis in 19 patients (36.5%), with a mean follow-up period of 23±11.8 months (median of 22.5 months, minimum of six and maximum of 49 months).

At the time of HCC diagnosis, the distribution of the Child-Pugh class was as follows: class A was indicated in 12 cases (23.1%), class B in 19 cases (36.5%), and class C in 21 cases (40.4%). Our utilization of the Child-Pugh score aligns with its validated effectiveness demonstrated in several prospective studies [26]. Furthermore, the prognostic assessment of the Child-Pugh score, comparable to the Model for End-stage Liver Disease (MELD) score, was affirmed in a recent systematic review [27]. Additionally, the Child-Pugh score was synergistically incorporated with the CLIP score for our study's encompassing evaluation of HCC prognosis. Despite the ongoing debate surrounding the choice of scoring systems for HCC, the CLIP score, recognized for its simplicity and relatively accurate prognostication, justified its application in our investigation [28]. This approach is endorsed by the American Joint Committee on Cancer and the American Hepatico-Pancreatico-Biliary Association [29].

Beyond prognostication, scoring systems play a pivotal role in guiding treatment considerations for patients. Notably, an increasing proportion of HCC patients have received therapy in recent years. However, this trend has been notably skewed towards non-curative therapies [30]. In our study, 59.6% of patients had a tumor deemed eligible for curative treatment based on size and multifocality criteria, yet only 32.7% underwent curative resection. Among those undergoing curative resection, tyrosine kinase inhibitors and immunotherapy with nivolumab were employed in 7.7% and 1.9% of cases, respectively.

In this investigation, elevated AFP levels exceeding 400 ng/mL, relapse, the presence of ascites, and a higher CLIP score demonstrated statistically significant associations with mortality. A meta-analysis by Zhang et al. [31] revealed that an AFP threshold of 400 ng/mL displayed lower sensitivity (32%) but heightened specificity (99%) compared to a lower threshold (i.e., 200 ng/mL). Toader et al. [32] further suggested a positive correlation between tumor burden and elevated AFP levels, even at a lower threshold of 200 ng/mL. However, Wang et al., in a propensity-matched analysis, did not establish a statistically significant association between AFP and metastasis in small tumors. Intriguingly, AFP failed to exhibit significance in predicting the prognosis of compensated cirrhosis [33].While the literature lacks a unanimous consensus on AFP's utility in HCC, it appears that relying solely on AFP for determining overall prognosis might be suboptimal. The presence of ascites, as observed in our study, emerged as a significant predictor of poorer outcomes [34,35]. This finding retained its statistical significance even after adjusting for other factors, including vascular invasion and tumor size [34]. Lastly, a higher CLIP score was associated with increased mortality in our study. Although our analysis did not establish an independent association of tumor morphological parameters or the presence of splenic vein thrombosis, the integration of these parameters in the CLIP score suggested a potential association, as supported by Hsu et al.'s study, which demonstrated increased mortality with a higher CLIP score [36]. Our analysis indicated that among patients with Child-Pugh class B, the risk of death was 1.21 times higher compared to those with Child-Pugh class A. Additionally, although not statistically significant, the risk of death was 2.81 times higher in patients with Child-Pugh class C compared to those with class A, aligning with findings reported by Hassan-Kadle et al. [37].

Several limitations stem from the retrospective design of this study. The modest sample size, compounded by attrition due to loss of follow-up or incomplete documentation, introduces potential bias and compromises the precision and generalizability of the study. Additionally, the exclusion of patients referred or managed in other institutions for various reasons, including further testing, patient preference, or treatment limitations, may render the current sample less representative of broader cohorts.

## Conclusions

Most cases of HCC in our patients were attributed to viral hepatitis, with the majority having liver cirrhosis. Higher mortality was noted among patients with elevated AFP >400 ng/mL, relapse, abdominal ascites, and a CLIP score. Targeted screening and health education should be advocated; in addition, social determinants should be proactively addressed.
